# Species integrity enhanced by a predation cost to hybrids in the wild

**DOI:** 10.1098/rsbl.2017.0208

**Published:** 2017-07-26

**Authors:** P. Anders Nilsson, Kaj Hulthén, Ben B. Chapman, Lars-Anders Hansson, Jakob Brodersen, Henrik Baktoft, Jerker Vinterstare, Christer Brönmark, Christian Skov

**Affiliations:** 1 Department of Biology—Aquatic Ecology, Lund University, Ecology Building, Lund, Sweden; 2 Department of Environmental and Life Sciences—Biology, Karlstad University, Karlstad, Sweden; 3 Ecology and Evolution Group, School of Life Sciences, University of Nottingham, Nottingham, UK; 4 Division of Evolution and Genomics, School of Biological Sciences, University of Manchester, Manchester, UK; 5 Department of Fish Ecology and Evolution, EAWAG Swiss Federal Institute of Aquatic Science and Technology, Center for Ecology, Evolution and Biogeochemistry, Kastanienbaum, Switzerland; 6 National Institute of Aquatic Resources, Technical University of Denmark (DTU), Silkeborg, Denmark

**Keywords:** predator–prey, cormorant, fish, diversity, evolution

## Abstract

Species integrity can be challenged, and even eroded, if closely related species can hybridize and produce fertile offspring of comparable fitness to that of parental species. The maintenance of newly diverged or closely related species therefore hinges on the establishment and effectiveness of pre- and/or post-zygotic reproductive barriers. Ecological selection, including predation, is often presumed to contribute to reduced hybrid fitness, but field evidence for a predation cost to hybridization remains elusive. Here we provide proof-of-concept for predation on hybrids being a postzygotic barrier to gene flow in the wild. Cyprinid fishes commonly produce fertile, viable hybrid offspring and therefore make excellent study organisms to investigate ecological costs to hybrids. We electronically tagged two freshwater cyprinid fish species (roach *Rutilus rutilus* and bream *Abramis brama*) and their hybrids in 2005. Tagged fish were returned to their lake of origin, exposing them to natural predation risk from apex avian predators (great cormorant, *Phalacrocorax carbo*). Scanning for regurgitated tags under cormorant roosts 3–4 years later identified cormorant-killed individual fish and allowed us to directly test for a predation cost to hybrids in the wild. Hybrid individuals were found significantly more susceptible to cormorant predation than individuals from either parental species. Such ecological selection against hybrids contributes to species integrity, and can enhance species diversification.

## 1. Introduction

The maintenance of species integrity requires low levels of interspecific gene flow. Gene flow between species can be limited by prezygotic barriers such as gametic and mechanical isolation, and/or postzygotic barriers such as hybrid inviability and infertility [1,2]. Yet examples occur in nature, particularly for closely related or newly formed species, where prezygotic barriers do not prevent hybrid offspring, where there is temporal and spatial overlap in breeding, and hybrid offspring are fertile. Ecological selection against hybrids is commonly invoked to explain the maintenance of species integrity [3–6], in this work defined as the prevention of species collapse into hybrid swarms. Ecological selection against hybrids may arise via mismatches between hybrid phenotypes and e.g. the environment or demands for anti-predator capacities, yet direct predation costs to hybridization in the wild remain inconclusive.

Predation is a ubiquitous and powerful ecological agent of natural selection [7–9] that may act as an extrinsic, postzygotic reproductive isolation barrier if invoking hybrid inviability [1]. If hybrids have intermediate phenotypes that fall between adaptive peaks occupied by parental species [10] hybrids may be removed by selection [11]. A higher probability of falling victim to predators for hybrids than parental species should provide an ecological mechanism enforcing species integrity. However, predator-mediated selection at the individual, phenotypic level is very difficult to document in nature, unless extraordinary prerequisites prevail, as shown in [9]. We here combine electronic tagging of two common freshwater fishes and their hybrids with a rare method of retrieving explicit records of individual predation events in the wild to evaluate a direct fitness cost to hybrid fish individuals due to predation by an apex avian predator.

## 2. Methods

**Figure 1.**
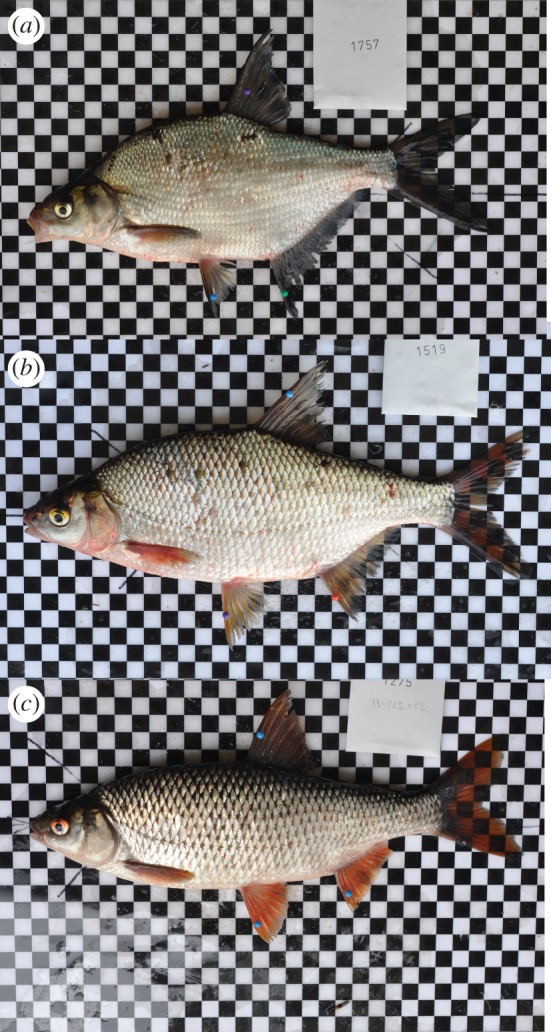
Photos of a bream (*a*, *Abramis brama*), a hybrid (*b*) and a roach (*c*, *Rutilus rutilus*) from Lake Loldrup. Discernible characters include scale size, fin colour, eye colour, length of anal fin, general morphology and body depth. (Photo Christian Skov.)

Members of the Cyprinidae have the highest frequency of hybridization among all groups of freshwater fishes [12]. Roach (*Rutilus rutilus*) and common bream (*Abramis brama*) are widely distributed and closely related freshwater cyprinid fish species that readily form fertile hybrids of distinctly intermediate body morphology (figure 1) [13–15]. The two species have external fertilization, are broadcast spawners with a preference for aquatic vegetation as spawning habitat and have temporal overlap of their spawning periods [16], which may facilitate hybridization between these species. We captured roach, bream and their hybrids in Danish Lake Loldrup and implanted individually coded electronic Passive Integrated Transponder (PIT) tags in all fish before releasing them back into the wild. The great cormorant (*Phalacrocorax carbo*) is a common fish-eating bird that preys upon tagged fish and regurgitates indigestible prey body parts along with tags at well-defined roosts and colonies on the lake [17,18]. After allowing 3–4 years of natural predation in the wild we used portable tag detectors to search below the roost and retrieve the unique identity codes from cormorant-killed individual fish.

The fish assemblage in Lake Loldrup (56°29′12.00 N, 9°26′42.33 E, surface area 0.39 km^2^, mean depth 1.2 m, maximum depth 3.3 m, mean summer Secchi depth 1.1 m) is numerically dominated by roach and bream, but also includes perch (*Perca fluviatilis*), pike (*Esox lucius*) and pikeperch (*Sander lucioperca*). We electrofished roach, bream and their natural hybrids (categorized by visual inspection according to morphological characters, total *n* = 456) between 30 September and 12 October 2005. Following capture we measured the total length (TL) of all individuals (hybrids: range 147–295 mm, 230 ± 26.2 mm (mean ± s.d.), *n* = 64; roach: range 125–295 mm, 181 ± 31.88 mm, *n* = 348, and bream: range = 135–294 mm, 248 ± 38.4 mm, *n* = 44. Next, we PIT tagged each fish (Texas Instruments, RI-TRP-RRHP, Plano, Texas, USA, half duplex, 134 kHz, 23.1 mm long, 3.85 mm diameter, 0.6 g in air) in the coelomic cavity through a vertical surgical incision (*ca* 5 mm) posterior to the left pelvic fin, a method with no observable effects on survival or body condition in cyprinid fish [19]. After recovery, fish were released at the capture location.

During 2008 and 2009 (i.e. 3–4 years after the study fish were tagged and released) we performed extensive scans for PIT tags at the cormorant roosting place by the lake and at a nearby cormorant nesting colony 5–12 km from the lake (depending on position in the study lake). We also scanned a more distant nesting colony (39 km away) and another roosting place (27 km away) for PIT tags on several occasions during the scanning period, but no tags were recovered at these locations. When scanning for tags, (systematically sweeping the whole area under roosts and colonies along predefined transects) operators used battery-powered (12 V, 7.2 A h) and portable flat-plate scanner systems with circular antennas (diameter 0.38 m) with four turns of 9-gauge plastic-coated (multistrand) oxygen-free copper wire, mounted on an antenna pole (length 145–160 cm), connected to a control module with a data logging memory (Texas Instruments Series 2000). Charge and read times were set to 25 ms. Once energized by the electromagnetic field generated by the antenna (read range 60–72 cm depending on tag orientation), a PIT tag transmits a unique identity code that is stored on the data logger.

Individual probability of falling victim to cormorant predation was estimated with a binary logistic generalized linear model, with cormorant predation (yes/no) as dependent variable predicted by factor fish type (bream, hybrid, roach) and individual body length at tagging (covariate), as well as their interaction term. Statistical analyses were performed using SPSS (SPSS Inc., Chicago, IL, USA), v. 23.0.

## 3. Results

**Figure 2.**
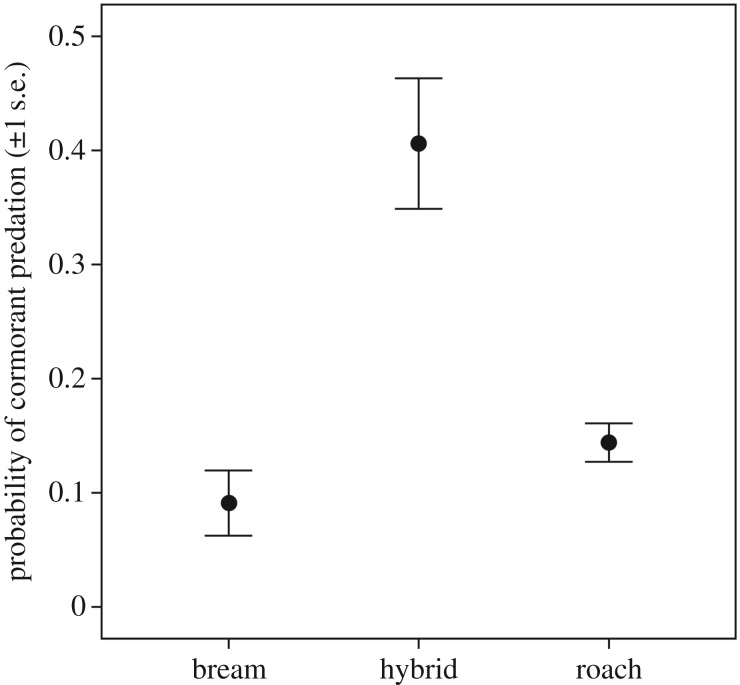
Roach × bream hybrids suffer a higher probability (estimated marginal means) of predation than their parental species when facing cormorant predators in the wild.

Our results [20] showed a higher probability of cormorant predation on, and thereby ecological selection against, roach × bream hybrids (figure 2). A total of 456 fish were tagged, of which 80 (17.5%) were predated by cormorants. Hybrids (initial *n* = 64) suffered higher predation (40.6%) than either roach (*n* = 348, 14.4%) or bream (*n* = 44, 9.1%). The full model (AIC = 268.496) was reduced by removing the non-significant interaction term (*p* = 0.488, Wald 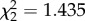), and the ensuing model (Akaike information criterion, AIC = 265.975) was reduced by removing the non-significant covariate body length (*p* = 0.374, Wald 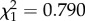), resulting in a final model containing only factor fish type as predictor (*p* < 0.001, Wald 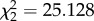, AIC = 264.748).

## 4. Discussion

Here we provide clear evidence of an elevated predation cost to hybrids relative to their parental species in a natural species complex of cyprinid fishes in the wild. While results from theoretical models and laboratory experiments have suggested hybrid disadvantage and the importance of ecological, postzygotic isolation for species integrity, only a few examples from natural populations of animals indicate predation as an important ecological selection agent [5,6,11]. Existing data are mostly descriptive. For example, populations of wild sticklebacks showed declining hybrid frequencies over age classes, suggesting but not mechanistically proving a fitness cost to hybrids [21]. In contrast, the results from our fish–cormorant–telemetry system allow us to directly link selection against hybrids to an identifiable agent of selection, substantiating the proposed idea that predation ecological selection contributes to the maintenance of species integrity and diversity in nature. The few available studies on predation as a postzygotic isolation mechanism have been performed under artificial conditions with inconclusive results. For example, studies have shown equal survival in hybrids and parent species in both sticklebacks [22] and tadpoles [23], and relatively higher hybrid survival in tadpoles [24], and in a *Daphnia* hybrid survival was higher compared with one parent species but lower than the other [25]. A single field experiment indicating a lower hybrid (tiger muskellunge, *Esox masquinongy* × *E. lucius*) survival than for parent species when exposed to predation from largemouth bass [26] was based on artificially reared and stocked fish. In our study of naturally spawned individuals in the wild, we clearly demonstrate the importance of avian predation as a fitness cost to roach × bream hybrid individuals, at overall cormorant predation rates comparable to other studies [27,28]. Both parental and hybrid species should be exposed to predation from other avian, mammal and fish predators, and even though we do not have data on their influence on overall mortality, we believe that efficient cormorant predators can contribute substantially to reduced success of hybrids. Moreover, the unlikely misclassifications of individual fish with morphologies intermediate between e.g. roach and hybrids would go both ways, and would not generate the higher probability for cormorant predation on hybrids seen in figure 2. Hybrids with phenotypes intermediate to their parental species should fall between phenotypic adaptive peaks. Phenotypic traits likely linked to anti-predator success of fish individuals in our study system include morphology and behaviour. A deep body morphology, as in bream, can incur benefits against gape-limited predation, whereas a shallow, fusiform roach shape should be hydrodynamically superior for high cruising speeds [29–31]. Hybrids have intermediate body morphology [15] (figure 1), which may incur an inferior anti-predator capacity. Anti-predator inferiority should be particularly imperative in light of the morphology-driven foraging success of roach × bream hybrids in cormorant-free novel environments [15], and our findings thereby contribute to the understanding of the evolution and maintenance of species integrity via ecologically enforced species barriers. Our results hereby highlight predation as an important selection force for species integrity, reducing species collapse from hybridization [32,33] and allowing species pair coexistence [22,34].

## Data accessibility

Data available at http://dx.doi.org/10.5061/dryad.h4513 [20].
